# Exploring the operational potential of the forest-photovoltaic utilizing the simulated solar tree

**DOI:** 10.1038/s41598-022-17102-5

**Published:** 2022-07-27

**Authors:** Dan-Bi Um

**Affiliations:** Korea Maritime Institute, Busan, South Korea

**Keywords:** Photovoltaics, Devices for energy harvesting, Energy policy

## Abstract

The aim of this study was to explore the operational potential of forest-photovoltaic by simulating solar tree installation. The forest-photovoltaic concept is to maintain carbon absorption activities in the lower part while acquiring solar energy by installing a photovoltaic structure on the upper part of forest land. This study was conducted by simulating solar tree installation using Google Earth satellite imagery in a mountainous area where an agrophotovoltaic system was already installed. When the simulation results were evaluated based on the installation guideline of the agrophotovoltaic system, it was confirmed that the operational potential of forest-photovoltaic was very high in almost all items of the guideline. Therefore, forest photovoltaic can be a possible alternative with priority in South Korea, where it is challenging to secure spatial competitiveness with a conventional flat fixed panel due to costly land prices. Although South Korea has been selected here as a case study, this discussion can be applied to other countries facing the disturbance risk to the forestry landscape due to solar power projects. To the best of the author's knowledge, this is the world's first study exploring the possibility of the forest-photovoltaic.

## Introduction

The biggest challenge facing humankind due to global warming is the fact that we have to radically reduce fossil fuels. Electric energy production and supply methods have been heavily debated for the past 10 to 20 years. Renewable energy, which does not burden the next generation, is emerging as a solution to tackle global warming. A previous study reports that it will be cheaper for South Korea to build new solar photovoltaic (PV) than to operate existing coal plants by 2025 to generate electricity for 30 years since the relative price competitiveness of solar energy will be improved compared to coal^1^. In this regard, solar power is expected to secure a position as the primary energy that replaces coal in the near future as the cost of energy production is radically decreasing with rapid technological development.

A solar tree has a structure replicating the branches and leaves of a natural tree^2,3^. Solar trees can produce more electrical energy than traditional flat fixed panels when placed in an equal amount of solar insolation for the same time duration^4–6^. The key element of the solar tree is to control the arrangement of solar panels so that sufficient sunlight can be irradiated to the lower forest cover. At the same time, the sunlight required for energy production is supplied in the upper part of the mountain^7^. The most significant advantage of the solar tree is that it occupies 1% of the land compared to the flat fixed panel by devising a holding system of PV modules with a vertical pole standing on the ground. For example, a 1 square meter in the case of a solar tree can generate approximately 5 kW power, whereas the traditional flat fixed panel requires 100 square meters^4^. Therefore, it could overcome the problem of land scarcity as solar trees can capture solar energy without deforestation in mountainous regions^8^.

70% of national land in South Korea consists of forests, about 20% of which are agricultural fields for food production, and the remaining 10% is made up of urban areas^9^. South Korea has the fourth highest forest ratio among Organization for Economic Co-operation and Development (OECD) member countries. UN Food and Agriculture Organization (FAO) recognized in its 1982 report that South Korea was the only country that had succeeded in forest restoration since World War II. Previous studies have analyzed changes in energy consumption behavior in the 1960s and 1970s as a decisive factor for successful forest reforestation in South Korea. As the economy grew rapidly until the 1970s, the decreased use of wood-based fuels could make it possible to control the rate at which trees were cut in the mountain^10^. Ironically, South Korea’s forests that have been successful in reforestation because of fossil fuels began to be damaged by solar energy plants in the last few years. As a result, 529 hectares were deforested in 2016, 1,435 hectares in 2017, and 2,443 hectares in 2018^11^. As the number of decades-old trees is felled due to the rapid increase of solar power plants in mountainous areas, secondary damages such as landslides and soil runoff are frequently occurring in South Korea.

The principle of the forest-photovoltaic is that the solar tree utilizes the remaining sunlight used for forest growth. The agrophotovoltaic system is a concept that produces crops and electricity in parallel in farmland and has recently been widely known as the solar sharing theory^12,13^. As the word itself means, the forest-photovoltaic system is to apply the concept of the agrophotovoltaic system to forests. A single hectare of a bamboo forest (6,200 plants) can absorb 35.5 tons of carbon dioxide every year. In South Korea, bamboo forests cover roughly 22,000 hectares of land, enough to absorb emissions from 150,000 households consisting of four members^14^. The forest-photovoltaic is to install a solar tree in such a forest area so that the forest can continue to absorb carbon while producing renewable energy. Compared to a general flat fixed panel, the solar tree has a higher structure and a stronger support base, increasing construction costs. As the demand for solar trees increases due to the development of new technology, more companies enter the market^4^. Therefore, it is expected that solar trees can be installed at a cost that can compete with the current flat fixed panel in the not-too-distant future.

Previous research on solar trees mainly focused on the configurative design to obtain energy efficiently^15^. Most of the recent discussions related to the solar tree mainly focus on issues verifying the efficiency of energy acquisition. In this process, previous studies comparing flat fixed panels and solar trees are observed^6^. It is a typically used method to evaluate the performance of a solar tree by mounting photovoltaic cells at various angles and heights. However, the individual solar tree-centered evaluation limits generalizing the research results in the forest-photovoltaic because there is a difference in performance depending on the solar tree sold in the market or the prototype manufactured by individual researchers. Although prior research provides valuable information on the performance of individual solar trees, there is a significant limitation in verifying the operational potential of the forest-photovoltaic by arranging solar trees in real mountainous areas. A previous study suggested using the solar tree in mountainous areas, which is closest to the topic covered in this study^8^. This study was conducted to explore the operational potential of the forest-photovoltaic by simulating solar tree installation using Google Earth satellite imagery acquired before solar power plant construction in a mountainous area where an agrophotovoltaic system is installed.

## Methods

### Experimental target

**Figure 1:**
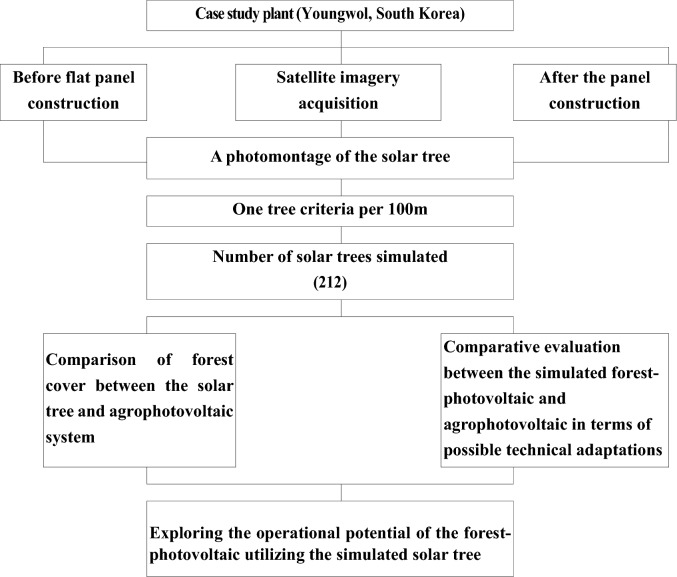
The procedures exploring the operational potential of the forest-photovoltaic.

**Figure 2:**
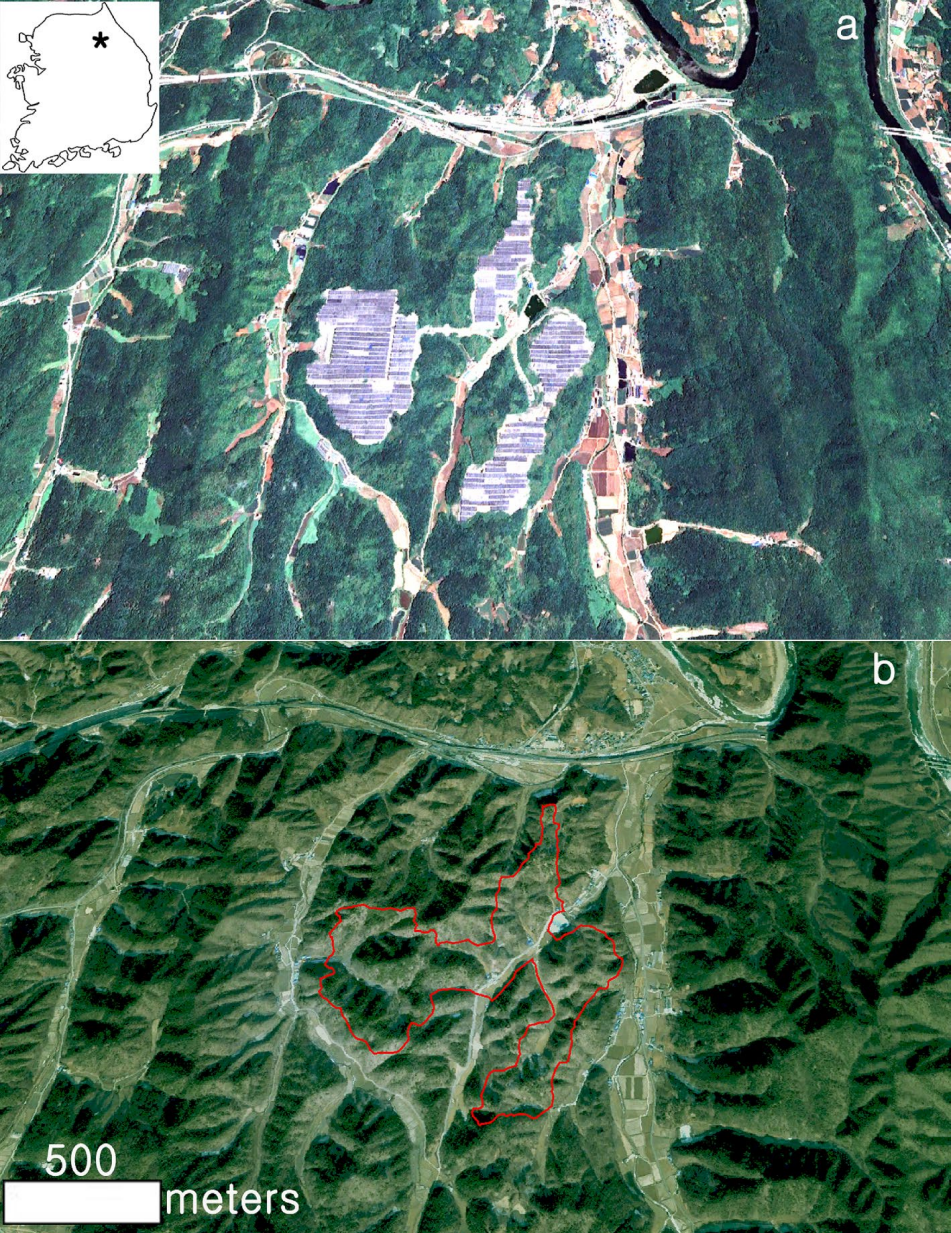
The study area (Youngwol solar power plant in Youngwol-gun, South Korea), (**a**) non-forestry landscape after flat fixed solar panel construction (Pléiades satellite imagery taken in July 2020). Inset: * represents the location of the study site in South Korea. (**b**) forestry landscape before the flat fixed solar panel (QuickBird satellite imagery taken in January 2005). The reddish polygon represents the coverage of solar plants in the upper figure (**a**). 3D magnified portion of the reddish polygon is displayed in Fig. 3, while 2D is presented in Fig. 5. Time series images (2005 and 2020) were obtained from Google Earth Pro 7.3.4 (https://www.google.com/earth/versions/#earth-pro). The map created in Google Earth Pro 7.3.4 and Adobe Photoshop CS3 (https://adobe-photoshop-cs3-update.en.softonic.com/).

A flowchart on the overall procedures of this research is presented in Fig. 1. The geographical location of the study area is in the east–west section of 128°23′22–128°24′27 longitude and the north–south section of 37°09′58–37°10′58 latitude (Yeondang-Ri, Youngwol county, South Korea). It is a mountainous area with a highly elevated plateau located at 400 m above sea level (Fig. 2). A modern slash-and-burn (the farming method that involves cutting and burning plants in a forest) is often carried out on the planation surface of a highly elevated plateau in summer. During the heavy rainy season in summer, the soil debris flowing into the river from the highland fields causes significant water pollution. In this regard, the highly elevated plateau has received considerable attention for its environmentally significant value^17^. The solar power plant was constructed by cutting a mountainous ridge available in the highly elevated plateau into flat land.

Yeongwol is the county that directed South Korea into an industrialized country, with the coal industry at the forefront in the 1960s. With the decline of the coal mining industry in the 1980s, the population, which once approached 110,000, including 8,600 miners, has decreased to 38,662 as of December 2020. Yeongwol, who led South Korea's modernization with fossil fuels, has sought opportunities to take another leap forward with the renewable energy business since the mid-2000s. The company Yeongwol Energy Station promotes that it is the biggest solar power plant combined with farmland in the world (capacity of 40 MW). 40 MW is equivalent to the total electricity used by the Youngwol-county inhabited by approximately 40,000 residents^18^. The solar panels installed on the 3 m high structure made a space for farming in the ground. One kind of ginseng, mountain garlic, is being grown in the space at the bottom of solar power facilities. In general, South Korea's photovoltaic power generation time is 3.3–3.5 h per day, but this solar farm has 3.7–4.1 h per day because it adopts highly advanced solar tracking technology that the PV panel moves according to the direction where the sunshine is strongly irradiated^18^. There is a difference between the project area (931,637m^2^) presented in the environmental impact statement^16^ and the area (1,100,872m^2^) occupied by the solar power plant measured by Google Earth (Table 1). It is considered that such a difference occurred because farmland, open space, and green area affected by this project were measured with one polygon, including small patches (for instance, green area preserved in its original condition) excluded from the project are presented in the environmental impact statement.

**Table 1:** Land use plan in environmental impact statement.

Category	Area (m^2^)	Composition ratio (%)
Power plant	602,695	64.6
Maintaining original land use	199,236	21.4
Greenery landscaping	129,706	13.9
Total	931,637	100

The plant has an installed capacity of 40 MW, equipped with approximately 130,000 solar panels. Maximum Power Pmax (W) per panel (300 W): Standard Test Conditions, STC, a cell temperature of 25 °C, and irradiance of 1000 W/m^2^ with an air mass of 1.5 spectra.

Source: 2016, environmental impact statement for Youngwol solar power plant^16^.

### Simulating solar tree installation

Google Earth Pro offers the most comprehensive geospatial database, including high-resolution imagery, historical imagery, street view, and points of interest. The 7.3.4 version of Google Earth Pro was utilized in the process of simulating solar tree^19^. Google Earth Pro provides satellite images taken at various times by region. For the study area, Digital Globe and Airbus^20^ offer high-resolution images (QuickBird: 65 cm, Pléiades: 50 cm). The data used in this study were fusion images combining panchromatic and multi-spectral images collected from Google Earth^21^. Google Earth Terms of Service for consumers offers a free download function and allows the use of images for research, but not mass downloads for commercial purposes^22^. This Terms of Service specifically mentioned that we love seeing creative applications of Google Maps, Google Earth, and Street View^23^. The latest Google Earth imagery for the study area was taken in July 2020 (summer). The most recent Google Earth imagery before the agrophotovoltaic plant construction was taken in January 2005. Therefore, the QuickBird acquired in January 2005 was used as the image before the construction of the agrophotovoltaic, while the Pléiades taken in July 2020 were utilized as the satellite imagery after the construction of the agrophotovoltaic plant.

Google Earth Pro on desktop offers mapping functions such as drawing lines, polygons, and 3D display of past and recent images^24,25^ The simulated solar tree should be presented according to the separation distance based on the same scale to secure a sense of presence in the field. Google Earth 3D image was used to reflect the size of the solar tree and the separation distance between solar trees, based on the realistic ground condition at eye level. Hanwha Q CELLS Korea donated a solar tree (4.8 × 4.1 m) to the National Assembly of South Korea on August 31, 2017 (Table 2). It is currently installed in front of the Parliament Building and operates to accumulate solar energy during the day and generates spectacular LED light at night using stored electricity^26^. The photomontage is a widespread method of representing landscape architecture in educational and professional settings^27^. A photomontage of the solar tree installed in the National Assembly was performed by utilizing Adobe Photoshop (version CS3) on the 3D image of the study area to visually reveal the situation before and after the solar panel installation. The slope distance was measured using the built-in elevation path measurement function in Google Earth Pro to arrange the solar tree at 100 m intervals in the three-dimensional image. Interpreting spatial objects in remote sensing is quantitatively to explore the information contained in the image according to the user requirement. Satellite images provided by Google Earth can be used to produce thematic maps through geometric pre-processing in image processing software^28^. In this study, a quantitative comparison for forest cover before and after solar tree installation was performed using the Google Earth images generated as a thematic map in Erdas Imagine (version 9.1). The forest area, solar panel, and open space were calculated using the polygon measurement function provided by Google Earth Pro to quantitatively evaluate changes in mountain landscape before and after solar tree installation.

**Table 2:** Detailed specification of a solar tree donated by Hanwha Q CELLS Korea.

Model	Type	Weight	Installation capacity
Q6LQUP4-G1.0	4BB Mono cell	2 ton	1.2 kw

In analyzing the operational potential of the forest photovoltaic, the most crucial step is to select the evaluation criteria for the project site. The analysis results are differentiated depending on which evaluation criteria are applied even to the same target. Analyzing the area-wide installation potential of the forest-photovoltaic raises the question of which variables and criteria should be used and how many variables should be used. These evaluation criteria can be presented in various ways depending on the sub-evaluation sector and the target area. One of the biggest problems in analyzing the forest-photovoltaic's operational potential is that it is difficult to quantify and objectively present evaluation results because they can be presented differently depending on the target area. So far, there is no internationally standardized evaluation system for the operational potential of the forest-photovoltaic.

Further, there have been no prior studies on realistically measurable variables to evaluate the operational potential of the forest-photovoltaic, focusing on solar trees simulated using Google Earth satellite imagery before agrophotovoltaic plant construction. In this study, the operational potential of the forest-photovoltaic was evaluated based on the checklist for possible technical adaptations used during the construction of an agrophotovoltaic plant, suggested by the Fraunhofer Institute for Solar Energy Systems in Germany^29^. However, it is judged that there is a limit to the reliability of the evaluation because the project characteristics unique to forest-photovoltaic (capturing carbon accumulated in the forest) are not sufficiently considered.

## Results

The most important step in simulating forest-photovoltaic solar trees is locating an installation point in a certain place based on specific criteria. There have been no prior studies on realistically measurable indicators such as separation distance and installation location in simulating forest-photovoltaic solar trees using Google Earth. However, not a road for vehicles, a path for hikers have already been built in the mountains. The area around the hiking trail is fragmented due to human intervention. Therefore, this hiking trail is the best place to install a forest-photovoltaic solar tree.

**Figure 3:**
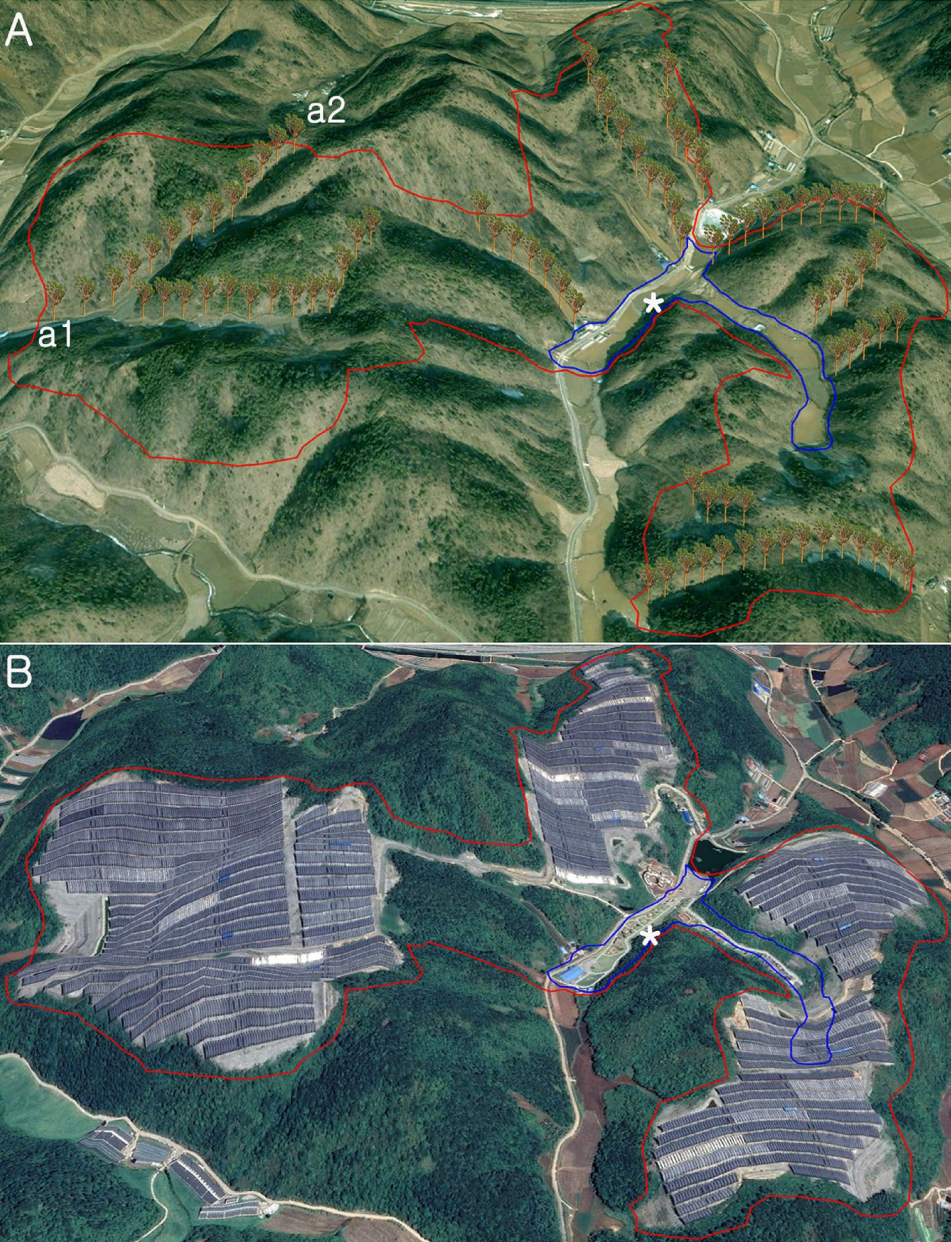
The forest-photovoltaic solar tree simulated a forestry landscape before flat agrophotovoltaic panel construction; see Fig. 2 for the location of the image. (**A**) Forest-photovoltaic solar tree simulation, a1-a2: example of hiking trail-based installation (slope distance: 1300 m), the total of 13 solar trees were installed based on one tree criteria per 100 m. (**B**) Damaged forestry landscape after the flat agrophotovoltaic panel installment: The reddish polygon represents coverage of solar plant while the blue polygon represents an agricultural field, including a farm road. A magnified portion of the blue polygon area is displayed in Fig. 4: * represents the location of the agricultural field at the study site. The scale bar is not inserted because the horizontal and vertical scales change depending on the viewing point in the 3D image. Time series images (2005 and 2020) were obtained from Google Earth Pro 7.3.4 (https://www.google.com/earth/versions/#earth-pro). The map created in Google Earth Pro 7.3.4 and Adobe Photoshop CS3 (https://adobe-photoshop-cs3-update.en.softonic.com/).

Each forest-photovoltaic project has its own engineering and environmental problems, which will depend on the size of the steel trunk and on-site characteristics such as topography, drainage, soil type, vegetation, and time of year when the work is carried out, etc. The most important factor is the separation distance between solar tree installation points since it will determine the range of disturbance. The separation distance between solar tree installation points can be presented in various ways according to the topographical characteristics of the project site. Comparing many mountain landscapes will help verify the effect of the separation distance among individual installation points. Since many variables such as elevation, slope, aspect, forest cover, and solar radiation affect the installation location, it is practically impossible to simulate the forest-photovoltaic solar tree considering everything. If the solar tree (4.8 × 4.1 m) is installed according to the scale of the 3D image, a considerable number of solar trees will be installed throughout the study area (1,100,872m^2^). For example, in the case of a solar tree placed on a hiking trail, the length of the trail is measured as 1300 m (a1-a2, in Fig. 3A). However, if too many solar trees are placed in a limited space, the solar tree displayed as too small in size caused significant limitations in terms of visual effect. When one per 100 m is arranged, the distribution of the solar tree can be remarkably visualized, so the simulation was performed based on one tree criteria per 100 m.

Figure 3 provides realistic evidence that forest-photovoltaic solar trees can be installed without destroying the mountain landscape. Unlike the satellite imagery taken in 2020, which completely lost its form as a mountain, the satellite imagery taken in 2005 installing solar trees confirms the evidence that the mountain landscape can be preserved as it is. In the satellite imagery taken in 2005 installing solar trees, the greenish landscape is identified, while the satellite imagery taken in 2020 shows the landscape structure replaced with a dark gray panel forming a heterogeneous landscape.

**Figure 4:**
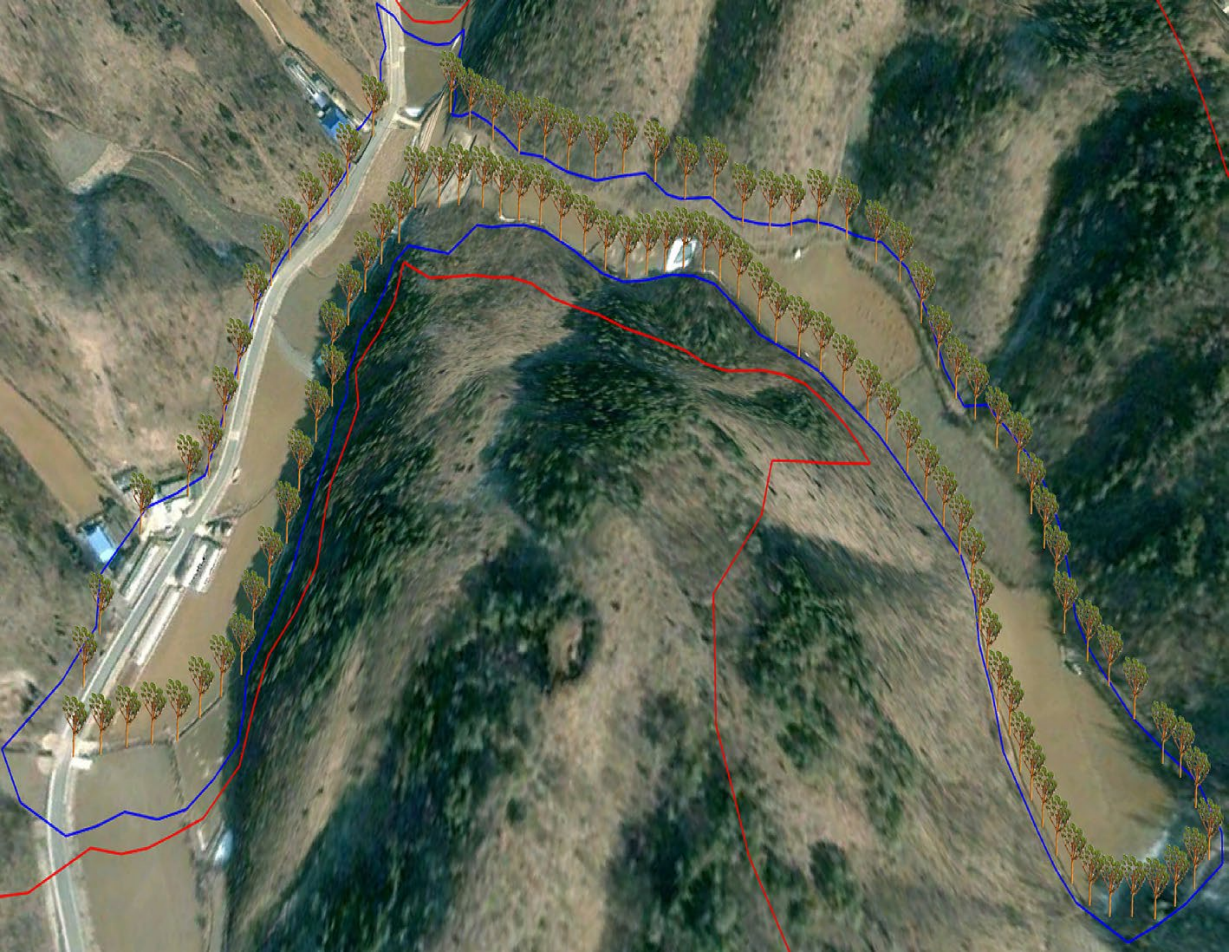
Solar tree installed around the space used as farmland (total: 114); see Fig. 2 for the location of the image. The satellite imagery was obtained from Google Earth Pro 7.3.4 (https://www.google.com/earth/versions/#earth-pro). The map created in Google Earth Pro 7.3.4 and Adobe Photoshop CS3 (https://adobe-photoshop-cs3-update.en.softonic.com/).

The separation distance of solar tree installation in farmland can be determined differently depending on the value judgment for the landscape impact caused by the forest-photovoltaic. A prototype landscape can be Korean rural society's shared values and consciousness for the natural environment formed by accommodating the local community's views^30^. Whether or not to install the solar tree based on the same separation distance (100 m) as the mountainous area for farmland is a process of seeking a compromise between the preservation of the prototype landscape and the utilization of solar energy. Farmland located on a highly elevated plateau should not be evaluated simply as the concept of land to cultivate crops. It is an essential component of a forest ecosystem that interacts with forests and exerts its function. The farmland existing in the experimental area has dual characteristics as forest edge and farmland. The simulation of mountain landscape can be divided into three different types, depending on the topography and mountain range: the separation distance (100 m) applied in the mountainous area, the separation distance applied in the farmland, and the separation distance in the area that intersects the farmland and the mountain area since agricultural land is at a lower elevation than mountainous areas and is already fragmented. Even if the solar tree is placed at a closer distance than in mountainous areas, it is not expected to disturb the surrounding mountainous ridge or skyline. In this regard, one solar tree was installed per 50 m separation distance (Fig. 4). As the separation distance between the solar trees was reduced in the farmland, a much larger number of solar trees (114) could be installed in the farmland compared to the mountain (88). However, the farmland area (58,070 m^2^) was smaller than the mountainous area (1,042,802m^2^, Table 3).

**Table 3:** Number of solar trees that can be installed.

Land cover	Area	Number of solar trees
Hiking trail-based (Fig. 3A)	1,042,802 m^2^	88
Around the space used as farmland (Blue polygon in Fig. 4: * mark, Fig. 3)	58,070 m^2^	114
Total	1,100,872m^2^	202

The latest Google Earth imagery for the study area before agrophotovoltaic plant construction was taken in January 2005 (winter). Therefore, a comparative evaluation was performed with the image (summer) taken in July 2020. There is a limitation to the objectivity in calculating the green area due to the seasonal difference.

**Figure 5:**
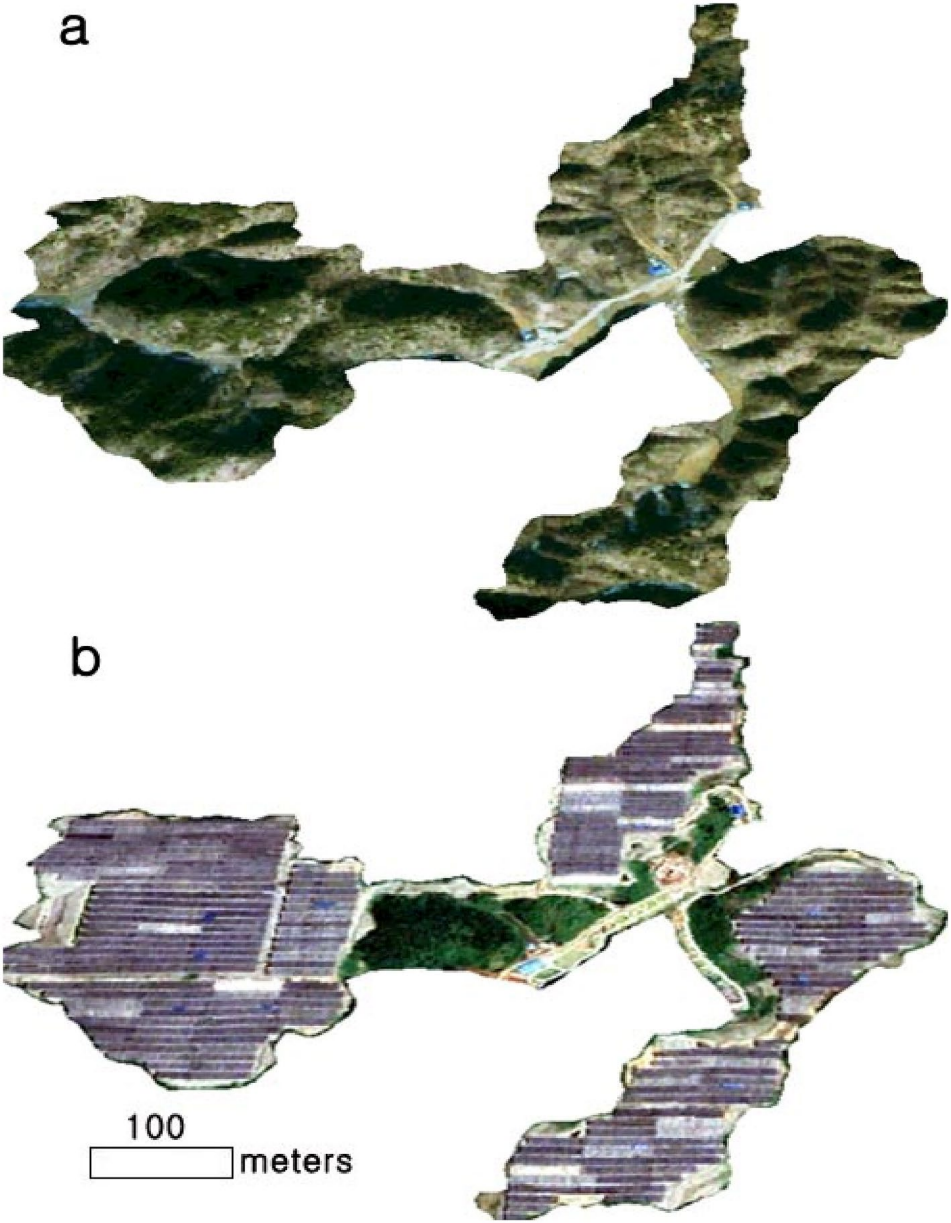
Comparison of forestry cover between the solar tree and agrophotovoltaic system, (**a**) forestry landscape before solar power plant construction (**b**) non-forestry landscape after agrophotovoltaic system construction. See Fig. 2 for the location of the image. Time series images (2005 and 2020) were obtained from Google Earth Pro 7.3.4 (https://www.google.com/earth/versions/#earth-pro). The map created in Google Earth Pro 7.3.4, Erdas Imagine 9.1(https://erdas-imagine.software.informer.com/9.1/) and Adobe Photoshop CS3 (https://adobe-photoshop-cs3-update.en.softonic.com/).

**Figure 6:**
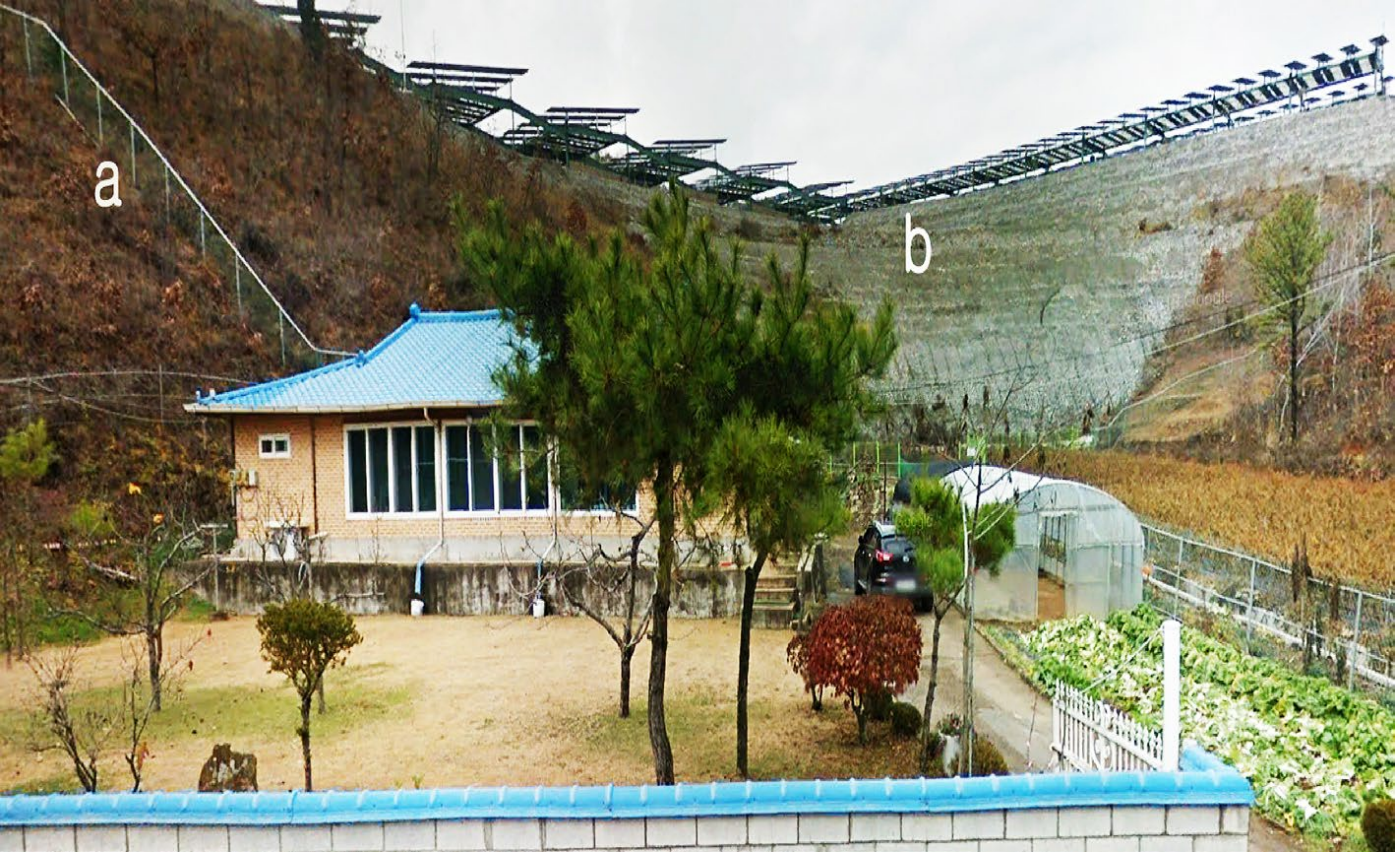
Typical scene of the agrophotovoltaic system observed in the experimental site, (**a**) a stair for farmers to access crops cultivated under solar panels, (**b**) cement-concrete structure converted from a mountainous landform. The fixed solar panel surroundings, captured from Google Earth street view, were obtained from Google Earth Pro 7.3.4 (https://www.google.com/earth/versions/#earth-pro). The image created in Google Earth Pro 7.3.4 and Adobe Photoshop CS3 (https://adobe-photoshop-cs3-update.en.softonic.com/).

The Google Earth imagery provides visual evidence that the project site has been thoroughly fragmented, confirming that 91% of the forest was converted into artificial man-made structures after the project (Fig. 5 and Table 4). On the other hand, it provides visual evidence that the typical natural scenery, such as green areas and highly elevated plateau, can be preserved in the solar tree simulation. Using satellite imagery, it is impossible to observe the mountain landscape under or around the solar panel where crops are cultivated in agrophotovoltaic plants. Satellite images provide information on the ground surface, but it is impossible to obtain information about the activities under the fixed solar panel. Figure 6 shows the scenery on the slopes around the fixed solar panel. The fixed solar panel and cement-concrete structure converted from a mountainous landform display a heterogeneous landscape in the mountain. Most of the environmental damage caused by installing agrophotovoltaic plants occurs on cutting slopes where forest cover has been removed. Therefore, it is desirable to minimize deforestation by applying vegetation engineering methods that help forests take root and prevent landslides. The advantage of agrophotovoltaic plants is that it is easy to respond to any malfunctioning in the power plant because there is a house where the farmers live right next to the power plant. For instance, the Energy Storage System (ESS) fire, which has become an issue recently^31^, could occur because the distance between the business site and the manager's place of residence is far away. In this project area, farmers can access crops grown under solar panels by stairs connected with the houses (Fig. 6a).

**Table 4:** Comparison of forest cover between the solar tree and agrophotovoltaic system (unit: m^2^).

	Solar tree	Agrophotovoltaic system
Forest cover (%)	1,042,802 (96)	73,604 (6.6)
Open space (%)	58,070 (4)	27,285 (2.4)
Artificial structures (%)	NA	999,983 (90.8)
Total	1,100,872m^2^ (100)	1,100,872m^2^ (100)

## Discussion

As Table 5 shows, most of the issues raised as problems in the operation of agrophotovoltaic can be improved by the solar tree. However, since the solar tree structure should firmly support the load of the upper photovoltaic module, it may be difficult to secure structural safety compared to the traditional method. The forest management is to increase carbon-capturing capability through forest gardening activities such as tree species renewal, timber extraction, harvesting, thinning, and pruning. A forest road is an essential infrastructure for cultivating the forest, collecting the produced timber, and preventing disasters such as forest fires^32^. Unfortunately, the density of forest roads in South Korea is 3.64 m/ha, far inferior to Germany (46 m/ha) and Austria (45 m/ha), advanced forestry countries^33^. Due to the huge government budget required for forest road construction, it is usual that forestry gardening is not being carried out in South Korea. This leads to the reality that deteriorates the value of forests as a carbon sink. For this reason, the density of accumulated forest per hectare is 165 m^3^/ha in South Korea^34^, which is less than half of advanced forestry countries (Germany: 321 m^3^/ha, New Zealand: 419 m^3^/ha).

**Table 5:** Comparative evaluation between the simulated forest-photovoltaic and agrophotovoltaic system in terms of possible technical adaptations.

Comparative elements*	Possible technical adaptations
What measures can be implemented to prevent an impairment of the forestry landscape?	Much better conditions than agrophotovoltaic
What are the wind and snow loads, and what are the additional loads due to the support height for the mounting structure?	Not confirmed by evidential information
Are the mounting structure and dimensions of the supports and carriers adapted to the working width of the forestry machinery? For example, should fenders be installed on the mounting structure?	No specific difference from the agrophotovoltaic
What is module technology to be used? The choice should always be adapted to the forestry use and products	No specific difference from the agrophotovoltaic
What local conditions, such as the field location, driving direction, headlands of the forestry machinery, or forest management systems, influence the modules' alignment?	Much better conditions than agrophotovoltaic
Is the use of storage batteries technically and economically reasonable?	No specific difference from the agrophotovoltaic
In which months do favorable, dry soil conditions exist to enable construction? When is the suitable field condition so that machines can lay ground slabs? This prevents soil compaction	Much better conditions than agrophotovoltaic
What anchoring or foundation system is to be used to ensure the removal of the system that leaves no trace?	Since it is installed as a semi-permanent facility, it will be much better conditioned than Agrophotovoltaic

*Source: Germany: Fraunhofer ISE (2021). Agrivoltaics: opportunities for agriculture and the energy transition. A guideline for Germany^29^.

If such a forest road is installed to enhance carbon absorption capacity, there will be a synergy of securing forest carbon and solar energy at the same time by installing solar trees around the forest road. Forest roads can shorten the construction period and reduce civil engineering costs in the forest-photovoltaic. In installing solar trees near forest road, basic maintenance such as ground compaction and leveling work could have been done around the road for a long time. Life cycle cost analysis usually considers initial construction, maintenance, and dismantling/disposal costs. There is no specific reason to dismantle the solar tree structure installed in the mountainous area. The solar tree should be constructed under the premise that it is operated as a semi-permanent facility such as transmission lines. Activities to capture carbon in forests require far less labor in terms of workload than cultivating crops. Crops need to be watered, fertilized to maintain soil fertility, and sprayed with pesticides to control pests, while forests require pruning or tree species renewal. For forests, pruning or tree species renewal once a year is sufficient, but crops require water, fertilizer, and pest control activities for each season or more. If solar trees can meet the installation standards for the agrophotovoltaic, it is judged that there will be no problem in achieving the operational requirements for acquiring forest carbon.

Prior research on solar trees focuses on the performance evaluation of energy acquisition by differentiating the height and angle of individual solar cells. It is judged that it has already been verified that solar trees can secure a much larger amount of solar energy at the experimental research level while occupying less space than the traditional flat fixed panel (Table 6). In addition, the ability to capture solar energy per unit area is superior to that of a flat fixed panel because panels installed in various directions can receive light energy from branches with several layers of panels^35^. However, it is hard to find out a previous study on whether such research at the laboratory level can be carried out in the real forest-photovoltaic, as in the experimental target area of this study. The prior research does not even suggest the idea of absorbing carbon and acquiring solar energy at the same time by installing a solar tree in a mountain area. Solar trees, simulated in Google Earth, presented visual evidence that the forest can absorb carbon from the atmosphere and make it possible to generate electricity at the same time. Solar tree-based forest-photovoltaic has a higher installation cost than agricultural photovoltaics since it has scattered distribution over a large area, although forest landscape can be preserved. However, it uses mountainous areas without deforestation, enables efficient national land use, and minimizes complaints such as opposition from residents regarding the installation site.

**Table 6:** Comparative evaluation of performance capturing solar energy between the forest-photovoltaic and agrophotovoltaic system.

Parameters	Forest-photovoltaic	Agrophotovoltaic system
Performance at capturing solar energy per unit area	More advantageous than flat fixed panels because these capture energy from several branches distributed at different altitudes	There is a limitation because the fixed panel captures solar energy only on one side parallel to the ground
Performance capturing solar energy according to incidence angle	Much more advantageous than flat fixed panels because the leaves can be oriented at different angles according to solar radiation	Same as above
Land space required for installation	Because panels are installed on a tower, the required ground area is much smaller than that required by flat fixed panels	Because panels capture solar energy from the ground, they require a much larger area than the solar tree
Cost of PV system	Costly due to complicated 3D design	Cheaper due to relatively simple design
Complaints from residents near the plant	Better than flat fixed panel	Worse than the solar tree

The Levelized Cost of Generating Electricity (LCOE) measures the average net present cost of electricity generation for a power plant over its lifetime. The land purchase cost is the most important parameter in calculating LCOE on the solar power plant in South Korea. South Korea is one of the most densely populated countries in the world^36^. A number of previous studies have pointed out a serious bubble in Korean land prices^37^. Compared to other countries, South Korea ranks third in the world in terms of land price^38^. So, purchasing the land is much higher than the money required to build a solar power plant. Since solar trees occupy a small area, they have considerable price competitiveness compared to flat fixed panels in countries with high land prices, such as South Korea. A previous study explores the relationship between electricity demand and country land area among the 42 world major countries^39^. South Korea is reported to be the lowest in the world because of its high electricity demand, while few land resources are available for solar power.

Table 7 presents the results of a nationwide survey in South Korea^40^ that the land purchase cost per unit of energy produced decreases as the size of solar power plant increases. This survey presents evidence that the solar power plant with a large-scale energy production capacity can secure the price competitiveness of LCOE. For example, if a solar power plant with a 3 MW installation capacity is built in a residential area, the land purchase cost is 1577 times that of the mountainous area. In the case of constructing a solar power plant with a 3 MW installation capacity in paddy fields, the cost of land purchase will be increased by 21 times compared to that of a mountainous area. This suggests that reducing the land purchase cost is the most important factor in ensuring the price competitiveness of LCOE for solar power in South Korea. When installing a solar power plant in South Korea, landowners can receive financial subsidies from the government, exempted from various land use regulations at the same time. The rapid increase in solar installations in South Korea reflects expectations for profits from rising land prices rather than energy sales^41^. This reality shows that South Korea is an attractive investment destination for the solar tree.

**Table 7:** Land purchase cost according to installation capacity of solar power plant (unit: Korean won/m^2^).

Land purchase cost	Installation capacity		
	100 kW	1 MW	3 MW
Forest (number of solar power plants)	8,686 (27)	3,518 (142)	1,700 (24)
Land on which new buildings can be built	1,138,828 (176)	1,403,803 (38)	2,680,700 (2)
Paddy field	119,235(24)	134,795(10)	36,100(1)

The solar tree has not been popularized yet, so the forest-photovoltaic field has many problems to be solved and is only in its infancy. The solar tree installed in mountainous areas will have a higher fixed load (self-load of solar power system), wind load, and snow load than the flat fixed panel. Previous studies evaluated fixed, wind, and snow loads according to AAMA 501.1–05 (Standard Test Method Using Dynamic Pressure) when constructing structures for various solar power systems (structure for folding solar power, external wall panel^42–44)^. Major module manufacturers do not yet have suitable solar tree modules for the forest-photovoltaic. The procedure for the solar tree to be commercialized has to deal with different international standardization and regulation schemes. Existing test standards provide detailed technical specifications focused on the flat fixed panel^45,46^. It was impossible to find a review of current regional and international standards dedicated to the solar tree. To enable solar tree technologies to successfully enter the market applications, it is crucial for the manufacturers to be able to prove liability in wind load and snow load operation of the solar tree. A follow-up study is needed to initiate legally binding international standards for solar trees' wind and snow load operation.

## Conclusion

This study is meaningful in presenting realistic evidence that the concept of an agrophotovoltaic system can be applied to perform carbon capture from forest land and energy production at the same time. Therefore, this study can be an objective reference to prioritize the forest-photovoltaic on solar power projects in the mountain landscape. Because of the perception that solar energy is eco-friendly compared to other renewable energy, subsidies are being paid for solar power installations in countries worldwide. Because of the solar subsidy policies of each government, an ironic situation occurs in which the forest cover as a carbon sink is destroyed while installing solar power in the mountainous area where land prices are relatively low. The New York Declaration on Forests adopted at the United Nations Secretary-General's Climate Summit held in New York in 2014 pledged to reduce the rate of deforestation by half by 2020 and restore hundreds of millions of acres of degraded land. Unfortunately, the international community, including South Korea, has failed to maintain this promise. The results of this study can serve as an opportunity for countries around the world to take action to stop deforestation, rather than making declarations that repeat old rhetoric.

## Data Availability

The datasets used and/or analysed during the current study are available from the corresponding author on reasonable request.
